# Effect of the environment on the secondary metabolic profile of *Tithonia diversifolia*: a model for environmental metabolomics of plants

**DOI:** 10.1038/srep29265

**Published:** 2016-07-07

**Authors:** Bruno Leite Sampaio, RuAngelie Edrada-Ebel, Fernando Batista Da Costa

**Affiliations:** 1 AsterBioChem Team, Laboratory of Pharmacognosy, School of Pharmaceutical Sciences of Ribeirão Preto, University of São Paulo. Av. do Café s/n, Monte Alegre, Ribeirão Preto, SP, 14040-903, Brazil; 2 Strathclyde Institute of Pharmacy and Biomedical Sciences, University of Strathclyde. 161 Cathedral Street, Glasgow, G4 0RE, Scotland, United Kingdom

## Abstract

*Tithonia diversifolia* is an invasive weed commonly found in tropical ecosystems. In this work, we investigate the influence of different abiotic environmental factors on the plant’s metabolite profile by multivariate statistical analyses of spectral data deduced by UHPLC-DAD-ESI-HRMS and NMR methods. Different plant part samples of *T. diversifolia* which included leaves, stems, roots, and inflorescences were collected from two Brazilian states throughout a 24-month period, along with the corresponding monthly environmental data. A metabolomic approach employing concatenated LC-MS and NMR data was utilised for the first time to study the relationships between environment and plant metabolism. A seasonal pattern was observed for the occurrence of metabolites that included sugars, sesquiterpenes lactones and phenolics in the leaf and stem parts, which can be correlated to the amount of rainfall and changes in temperature. The distribution of the metabolites in the inflorescence and root parts were mainly affected by variation of some soil nutrients such as Ca, Mg, P, K and Cu. We highlight the environment-metabolism relationship for *T. diversifolia* and the combined analytical approach to obtain reliable data that contributed to a holistic understanding of the influence of abiotic environmental factors on the production of metabolites in various plant parts.

Specimens of same plant species growing under different environmental conditions show significant differences in the production and accumulation of the primary and secondary metabolites^1^,^2^,^3^,^4^,^5^,^6^,^7^,^8^. Influenced by environmental factors, respective group of secondary metabolites act as a chemical interface between the plant and its environment. The chemical interaction between plants and their environment is mediated mainly by the biosynthesis of secondary metabolites, which exert their biological roles, as a plastic adaptive response to their environment. Such chemical interaction often includes variations in the production of plant metabolites^1^,^2^,^3^,^9^,^10^,^11^,^12^. Therefore, the study of these variations is very useful in the chemical characterization of plants of the same species which are collected from different regions and this is when the different geographical origin of a plant material is taken into account^13^,^14^,^15^. Some processes can be the main sources of variation in the levels of metabolites for individual plant species. These processes involve long term acclimation or local adaptation, seasonal differences related to phenology or environmental changes in the biotic and abiotic factors, geographical differences involving different populations (genetic differences within a plant species), or different environmental conditions of the growth location of the species individuals, especially when they have genetic homogeneity (i.e. cultivars and/or clones)^16^,^17^.

Metabolomics is an interesting approach that has been used in the plant sciences, especially in ecological studies, in investigating the effects of environmental factors on plant metabolism. Metabolomic profile data have been utilised to compare different species from the same family, or individuals from populations from the same species growing under different environmental conditions, or changes in metabolite production of individuals within the same population at different seasons^18^,^19^,^20^,^21^.

*Tithonia diversifolia* (Helms.) A. Gray, commonly known as tree marigold or Mexican sunflower, is a perennial herb from the family Asteraceae, tribe Heliantheae, native to both Mexico and Central America. *T. diversifolia* is an interesting example or representative of a plant species that can adapt to different types of environment. It is widely distributed around the world, mainly in tropical and sub-tropical areas of America, Africa and Asia^22^,^23^,^24^. *T. diversifolia* has been described as an invasive weed that can establish itself in different types of ecosystems^25^,^26^,^27^. This plant species has been associated with ecological imbalance. Due to its high adaptability to different environmental conditions (climate and soil), high dispersion and rapid growth rate, *T. diversifolia* has an advantage to occupy degraded areas and dominate over native species^26^,^28^.

The chemical constituents of *T. diversifolia* have been well described in the literature. Most of these reported compounds belong to the class of sesquiterpene lactones, flavonoids^23^ and *trans*-cinnamic acid derivatives (mainly caffeoylquinic acid)^29^. The major occurrence of hydrocarbon mono- and sesquiterpenes, especially α-pinene and β-caryophyllene are essential oils commonly found in leaves and inflorescences of *T. diversifolia*^24^. Several studies in recent years have shown that extracts and secondary metabolites isolated from leaves and inflorescences of *T. diversifolia* exhibited interesting biological activities such as anti-inflammatory^30^,^31^, antimalarial^32^, cytotoxic^33^, gastro-protective^22^, antimicrobial^34^,^35^, chemopreventive^36^ and antihyperglycemic^37^,^38^.

Due to the wide-distribution and great adaptive response capability of *T. diversifolia* to different environmental conditions, and its proficiency to yield potential therapeutic natural products, a specific and innovative metabolomic approach was used to obtain the respective chemical profiles of the samples and to correlate them with the environmental data (climate and soil) from distinct regions at different seasons. The metabolomic approach is useful to understand how variations in plant metabolism can be a response to changes in the surrounding environmental conditions and to be able to propose a statistically sound experimental model that can be applied to environmental metabolomics of plants in the fields of ecology, agriculture, medicinal plants research and other related fields of research.

Considering the ecological adaptability features of *T. diversifolia* as an invasive weed, this species was proven as an adequate object of study to establish an experimental model for environmental metabolomics of plants through a new concatenated approach combining UHPLC-DAD-(ESI)-HRMS and NMR spectral data. Mass spectrometry has the advantage of detecting metabolites at micro- to nanogram concentrations while NMR provides more information on the identity of the metabolites. The new concatenated approach was an efficient method in directly matching the mass to ratio data with the structure of the respective compounds which can only be specified from the NMR datasets that expedites the dereplication step. This approach was used for the first time to carry out a comparative study of different plant part samples of specimens obtained from two environmentally diverse regions of Brazil and collected at different seasons throughout a 24-month period. For this study, metabolomic profiling was focused on a set of compounds that correspond to the main classes of secondary metabolites encountered in *T. diversifolia*, particularly sesquiterpene lactones and phenolics, which are related to the most relevant biological properties presented by this species; in addition, primary metabolites also compose the set of analysed compounds. This study is strategic in provision to bioprospecting the worthwhileness of invasive weeds in yielding bioactive natural products at different environmental conditions. A regular future harvest of such invasive weeds for bioprospecting purposes will maintain ecological balance of the natural home species.

## Results

### Environmental data

The environmental data (climate and soil parameters) were obtained during the 24 months of the study. The obtained climate (see Fig. S1) and soil (see Tables S1 and S2) data are shown under Supplementary Information.

### UHPLC-DAD-(ESI)-HRMS and NMR data

Analyses and data processing of 170 extracts by UHPLC-DAD-(ESI)-HRMS recorded 1,277 and 1,084 peaks in the positive and the negative mode, respectively. The negative mode results were used as a basis for proposing the groups for variation analysis of *T. diversifolia* metabolites. On the other hand, the processed ^1^H and *J*-resolved NMR spectral data were also subjected to PCA and OPLS-DA. Results of the multivariate analysis of the UHPLC-DAD-(ESI)-HRMS and *J*-resolved NMR spectral data were complementary.

**Figure 1:**
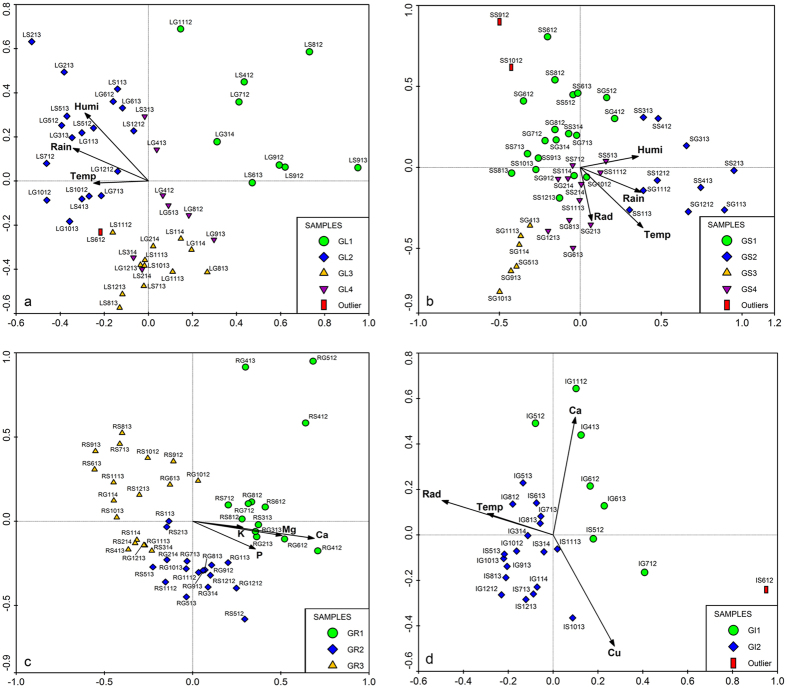
PCA score scatter plots of the concatenated data exhibiting the correlation of the environmental factors with the occurrence of the metabolites from various plant part extracts of *T. diversifolia:* leaves (**a**), stems (**b**), roots (**c**) and inflorescences (**d**). Legend: G = Group; L = Leaves; S = Stems; R = Roots; I = Inflorescences; Number (eg. 112 for Jan 2012 or 1114 for Nov 2014) represents the month (1 for January until 12 for December) and year (12 for 2012 until 14 for 2014); Humi = Humidity; Rain = Rainfall; Temp = Temperature; Rad = Solar radiation; Ca = Soil calcium; Mg = Soil magnesium; P = Soil phosphorus; K = Soil potassium; Cu = Soil copper.

**Figure 2:**
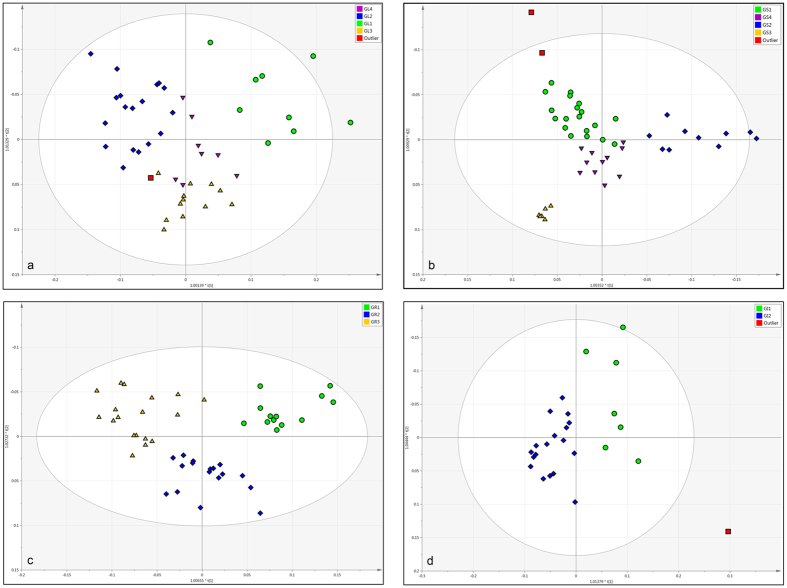
OPLS-DA score scatter plots of the concatenated data obtained from various plant extracts of *T. diversifolia*: leaves (**a**), stems (**b**), roots (**c**) and inflorescences (**d**). G = Group; L = Leaves; S = Stems; R = Roots; I = Inflorescences.

The high-resolution MS and NMR spectral data were concatenated^39^ and subjected to the same multivariate methods of analyses and strategies. It was observed that all acquired results from the different ways of data analyses (using separate or concatenated data) were very similar. However, multivariate analyses of the concatenated data gave better separation of the proposed groups which could further demonstrate and explain the relationship between the occurrence of the metabolites in *T. diversifolia* and the abiotic environmental factors. The dendrograms obtained by HCA analysis of the respective crude extracts of leaves, stems, roots and inflorescences of *T. diversifolia* as well as the clustering proposal are shown under Supplementary Information (see Figures S2 to S5). PCA and OPLS-DA score scatter plots of the concatenated data of various plant parts according to different environmental conditions are shown in Figs 1 and 2, respectively.

**Figure 3:**
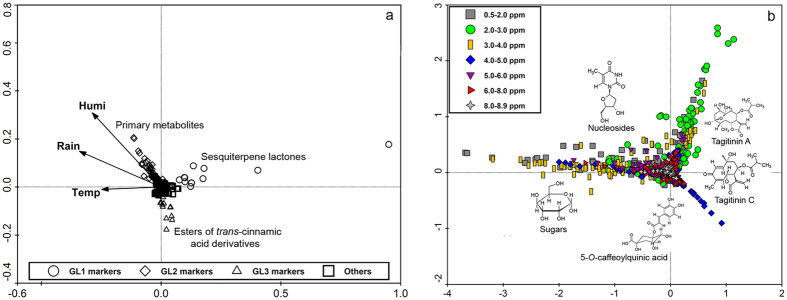
PCA loading plots of the concatenated data (**a**) and *J*-resolved chemical shift data (**b**) of *T. diversifolia* leaf extracts to correlate discriminant classes of metabolites with the various environmental factors. G = Group; L = Leaves; Rain = Rainfall; Humi = Humidity; Temp = Temperature.

**Figure 4:**
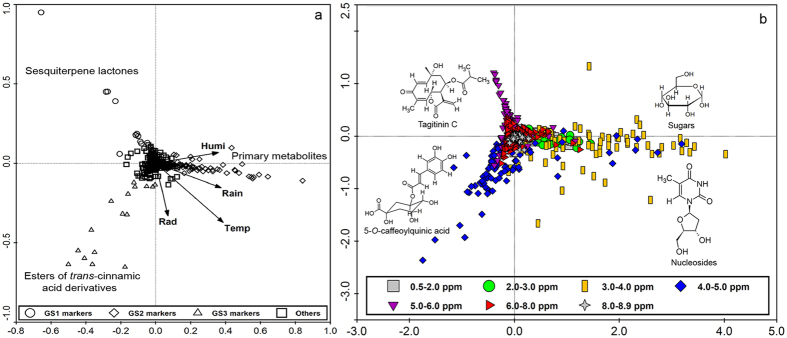
PCA loading plots of the concatenated data (**a**) and *J*-resolved chemical shift data (**b**) of *T. diversifolia* stem extracts to correlate discriminant classes of metabolites with the various environmental factors. G = Group; S = Stems; Rain = Rainfall; Humi = Humidity; Temp = Temperature; Rad = Solar radiation.

**Figure 5:**
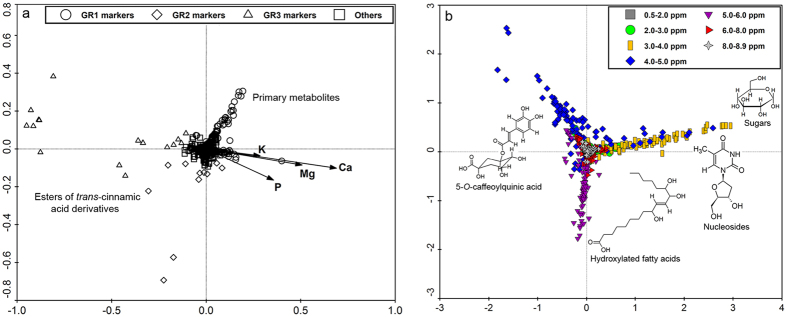
PCA loading plots of the concatenated data (**a**) and *J*-resolved chemical shift data (**b**) of *T. diversifolia* root extracts to correlate discriminant classes of metabolites with the variations in soil nutrients. G = Group; R = Roots; Ca = Soil calcium; Mg = Soil magnesium; P = Soil phosphorus; K = Soil potassium.

**Figure 6:**
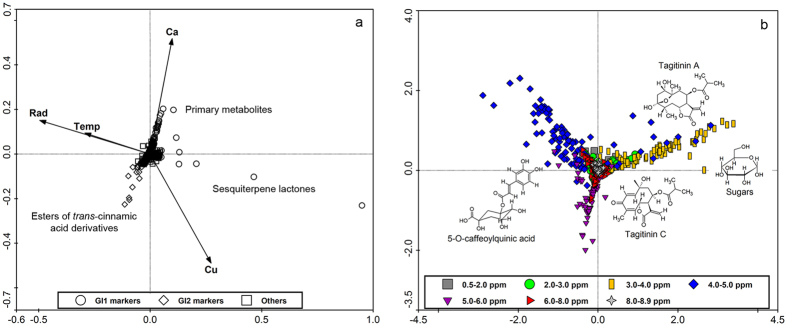
PCA loading plots of the concatenated data (**a**) and *J*-resolved chemical shift data (**b**) of *T. diversifolia* inflorescence extracts to correlate discriminant classes of metabolites with the various environmental factors. G = Group; I = Inflorescences; Ca = Soil calcium; Cu = Soil copper; Temp = Temperature; Rad = Solar radiation.

S-line analyses of the *J*-resolved spectral data sets of respective proposed groups (see Figures S6 to S9) provided characteristic chemical shift signals for each group of samples of *T. diversifolia* which can be identified with standard reference compounds previously isolated from this plant. These characteristic chemical shifts from the crude extracts were used in multivariate analyses to provide quick qualitative information to relate the occurrence of respective metabolites in coherence with environmental changes. The PCA loading plots of the concatenated and *J*-resolved data for the respective plant parts are shown in Figs 3, 4, 5, 6. Dereplication identified a class of 55 major metabolites used as discriminants in the multivariate analysis (Table S3).

## Discussion

Based on the results, it is possible to predict and hypothesize the chemical features of *T. diversifolia* in rationale with changes in environmental factors by taking into account the variations in metabolic profile perceived through the occurrence of major metabolites in various plant parts. Regarding the metabolic composition, considering the role of the genetic differences on the production of metabolites in *T. diversifolia*, the obtained results, especially for leaf and stem samples, showed that the genetic differences between the two groups of matrices do not seem to be as significant as the changes in environmental conditions. When analysing the clusters projected for leaves (Figs 1a and S2) and stems (Figs 1b and S3), it can be observed that samples of two different groups of matrices were clustered together, so that the main common feature between samples within the cluster is the time of the year when the samples were collected. It could be also highlighted that, even for the roots and inflorescences samples, it was observed that the main pattern of separation between the clusters was not based on the geographical origin of samples, but on the seasonal variation of the environmental factors, so that, biochemically, samples were not grouped primarily according to the group of matrices of which they were obtained. Therefore, the results of this study certainly added new and relevant information on the seasonal variation of the major classes of metabolites found in *T. diversifolia*.

The environmental factors monitored in this study include temperature, rainfall, humidity and solar radiation as well as the amount of soil macro- and micronutrients which proved to correlate to variations in the sugar and nucleoside content as well as secondary metabolite composition of *T. diversifolia*. It should also be kept in mind that other environmental factors that may affect soil conditions in the two remotely distant regions have not demonstrated significant influence on the metabolic profile of the plant.

An interesting aspect that can be observed is that the environmental factors do not seem to exert a constant effect on the entire metabolism of the plant, so that each respective plant part responds differently to changes in environmental conditions. This difference in the metabolic response according to the plant parts has been described in a comparative study of the metabolism of *Holcus lanatus* L. and *Alopecurus pratensis* L. (Poaceae). Differences in the accumulation of primary and secondary metabolites between the shoots and roots of both species in different seasons and conditions of drought were observed through LC-MS and NMR spectral data obtained for the samples^40^.

Based on the results of the PCA and HCA performed on the concatenated data obtained by UHPLC-DAD-(ESI)-HRMS and *J*-resolved NMR analyses, it was possible to propose groups in agreement to the sample’s metabolic profiles in relation with the accrued environmental data for the respective plant part as follows: leaf samples yielded four groups with one outlier (Fig. 1a), stem samples were grouped into four with two outliers (Fig. 1b), root samples indicated three groups (Fig. 1c) while inflorescences resulted to two groups with one outlier (Fig. 1d). The grouping proposal of the various *T. diversifolia* extracts (Fig. 1) showed that, variation in metabolic profile in the leaves and stems seems to be related mainly on rainfall and humidity levels, with temperature and solar radiation also exerting some influence on the metabolic profile. Inflorescences and roots were grouped according to the availability of certain nutrients in the soil, especially soil macronutrients (Ca, Mg, P and K) and the micronutrient Cu, with solar radiation and temperature also significantly affecting the metabolic profile in the inflorescences.

Several climate factors such as water availability, temperature and solar radiation are well described as being able to influence the production of metabolites^2^,^21^,^41^,^42^,^43^. Thus, plants under conditions of stress induced by climate factors (i.e. drought, high temperatures, freezing, wide thermal amplitude, high levels of solar radiation) may show changes in the production of different metabolite classes.

Specimens of *T. diversifolia* used for this study were cultivated in a phytogeographic domain indicated as the Brazilian Cerrado, a floristically diverse savannah covering approximately an area larger than two million km^2^ of Central Brazil, representing about 23% of the territory of the country (the same size as Western Europe)^44^,^45^. The predominant climate in the Brazilian Cerrado is semi-humid tropical, which is characterized by strong seasonality of rainfall, with two distinct seasons: the dry season that typically extends from May to September and a rainy season from October to April, with an annual average temperature ranging from 21.3–27.2 °C^46^.

The availability of water is a known factor related to the variation in the production of metabolites in plants^2^,^21^. Specimens used in this study were grown in places that have a patchy distribution of rainfall throughout the year. Analyses of the PCA plots determined the relationship between the seasonality of the rain in the area and the metabolic profile of the leaves and stems (Fig. 1a,b). It can be observed that samples collected during the rainiest periods tend to cluster and were rich in primary metabolites such as sugars and nucleosides. Leaf and stem samples collected during the drier periods were rich in secondary metabolites such as sesquiterpene lactones particularly in the cold months while there was an increase production of *trans*-cinammic acid ester derivatives during the hot months. Collected samples of leaves and stems during the transition period between the dry and rainy seasons yielded intermediate levels of both primary and secondary metabolites.

In accordance to the PCA (Fig. 1c,d) and OPLS-DA (Fig. 2c,d) plots, root and inflorescence samples of *T. diversifolia* were grouped in a pattern related mainly to the availability of certain soil nutrients. For inflorescences, temperature and solar radiation also seem to influence metabolite production. As clearly shown in Figs 1c and 2c, root samples were distributed into three large groups. The first group, designated as GR1, composed mostly of samples from the state of Goiás which were mainly collected in the first year of study and samples from state of São Paulo were collected in the first half of the first year of study. During this collection period, there were higher levels of soil macronutrients in both regions and GR1 was characterized by lower accumulation of secondary metabolites. The second group, GR2 consisted of a larger number of samples from Goiás, mainly collected in the second year of the study, and samples from São Paulo collected in the second half of the first year. The observed levels of soil macronutrients decreased during this period and in parallel, GR2 samples indicated low but significant accumulation of phenolic compounds. For the third group, GR3, composed mainly of samples from São Paulo collected during the second year of study, it was observed the lowest levels of soil macronutrients and, consequently, the high accumulation of phenolic compounds, especially esters of *trans*-cinnamic acid derivatives, in the group samples.

For the inflorescences, it was possible to observe the existence of two main groups (Figs 1d and 2d). Group GI1 was primarily composed of samples from Goiás, where the soil showed higher levels of Ca, and the temperature and levels of solar radiation were low during the flowering season. The accumulation of sugars was observed along with high Ca concentration in the soil, while, the occurrence of sesquiterpene lactones was correlated with the increase of Cu levels. Group GI2 consisted mostly of samples collected in São Paulo described to have higher temperatures and higher levels of solar radiation. The collected inflorescences indicated the accumulation of esters of *trans*-cinnamic acid derivatives. Similar to the collection from Goiás, the presence of certain soil nutrients can also be associated with the occurrence of some metabolites. Moderate levels of primary metabolites and sesquiterpene lactones were detected in inflorescence samples of plants from soils rich in Ca or Cu, respectively.

The four aerial part outliers (one for the leaves, two for the stems and one for the inflorescences) were characterized by high accumulation of two specific sesquiterpene lactones: namely tagitinin A but mainly tagitinin C. All these outlying samples were collected from São Paulo during the dry season of the first year of study. The increased accumulation of tagitinins A and C was observed to be related to a specific combination of environmental conditions, in this case, low water availability, low temperatures and low levels of solar radiation.

The PCA loading plots obtained from the NMR (*J*-resolved) data showed signals with chemical shift values representing major classes of metabolites used as discriminants for the respective proposed groups. For example, as shown in Figs 3 and 4, resonances between 5 to 6 ppm were related to olefinic double bonds present in an aliphatic carbon skeleton while those between 2 to 3 ppm corresponded to hydrogens attached to a saturated carbon in the environment of a carbonyl group typical to unsaturated fatty acids and sesquiterpene lactones, as found in tagitinin C in samples of leaves and stems grouped under GL1 and GS1, which were characterized by the accumulation of these substances.

The characterization of groups rich in esters of *trans*-cinnamic acid congeners was not so clear by using only the NMR *J*-resolved data results because of overlapping resonances belonging to different classes of metabolites, such as signals between 6 to 7 ppm for phenolic hydrogens which overlap with hydrogens of conjugated double bonds present in some type of sesquiterpene lactones. The use of the UHPLC-DAD-(ESI)-HRMS data were very useful to discriminate both groups. On the other hand, the NMR *J*-resolved data were very useful in characterizing groups of samples rich in sugars indicated by proton resonances from 3 to 5 ppm typical for hydroxylated methine protons common in carbohydrate molecules. The OPLS-DA S-line plots obtained from the NMR *J*-resolved data, shown in the Figures S6 to S9 (supplementary information), illustrate how some ^1^H-NMR signals from different classes of metabolites can be useful to separate two different groups of samples within the same species, as a good discriminant between diverse chemical profiles (Figs 3, 4, 5, 6).

The mechanism involved in the influence of climate factors on *T. diversifolia* metabolism appears to be related to seasonal induction of stress factors in the plant, such as: (i) drought stress, which can cause a reduction in photosynthetic rate with a consequent increase in production of reactive oxygen species (ROS), therefore resulting in an increase in the production of phenolic compounds (natural antioxidants) during the dry season as a defense mechanism^2^,^21^,^47^; (ii) thermal stress caused by large temperature variation range during the year, especially during drier periods, which can affect metabolic regulation, permeability to water and CO_2_ and the rate of intracellular reactions^2^,^48^ as well as improve the antioxidant properties with an increase availability of carbohydrates^48^; and (iii) stress by solar radiation, particularly UV-B radiation (280–320 nm), which mainly affects the production of phenolic compounds (tannins, anthocyanins, flavonoids and other *trans*-cinnamic acid derivatives) which, besides contributing to absorption and/or dissipation of solar energy, provide protection against the deleterious effects of UV radiation, such as tissue damage induced directly by UV-B or formation of free radicals and other oxidative species^49^,^50^,^51^.

The influence that the soil nutrients Ca, Mg, P, K and Cu exert on the roots and inflorescences of *T. diversifolia* can be related to several physiological roles that such nutrients play in higher plants, since these nutrients are essential for normal growth and functioning of metabolism^52^. The accumulation of esters of *trans*-cinnamic acid derivatives in roots from soils with lower levels of macronutrients may be a response to the reduced nutrient availability, since polyphenols bound to sesquioxides have the ability to prevent adsorption of phosphate. Moreover, phenolic acids also have the ability of desorb the bound phosphate, thus increasing the availability of inorganic P in the soil, aside from the fact that phenolic compounds can also retain inorganic exchangeable cations (Ca, Mg and K) providing sorption sites on acid soils^53^.

Considering all the results presented herein, we can highlight the relationships between environment and the metabolic profile of *T. diversifolia*, in which the variation in the production of certain classes of metabolites in the plant appeared to be a direct response to changes in conditions of its surrounding environment. It can also be highlighted that the set of analytical techniques selected for this work combined with proper multivariate analysis allowed us to obtain statistically-sound, reliable data that contribute to a holistic understanding of how some primary but mainly secondary metabolism are affected in the species according to the changes of the abiotic environmental factors of the location where it can be encountered and how respective plant parts is differently affected by the environment.

There are different works reporting the use of a metabolomic approach to study the influence of the environmental factors on the metabolite production of some economically important plants, such as rice cultivars^54^, genetic modified maize^55^,^56^,^57^, citrus rootstocks^58^ and green tea^59^. However, for the first time, metabolic information was obtained by the fusion of two data sets originated from high resolution LC-MS and NMR, to perform the statistical analyses. The fused concatenated data set was an efficient method in directly matching the *m/z* signals with the structure of the respective compounds within a cluster which expedites the dereplication step.

Thus, the approach reported herein may be useful in the study of other invasive and highly adaptable species as well as in the study of the effects of environmental changes in the development of plants, which can also be extended to explore quality control or biological properties of further economically important crops, food species or medicinal plants, the latter responsible for the accumulation of active substances that exert pharmacological effect in humans.

## Methods

### Collection of plant material

For this study, samples were collected in condition ex situ from two groups of matrices, each composed of 48 individuals, cultivated in two different states of Brazil, one located in the Garden of Medicinal Plants of the School of Pharmaceutical Sciences of Ribeirão Preto, University of São Paulo, Ribeirão Preto, state of São Paulo (latitude 21° 10′ 07.4″ S, longitude 47° 50′ 49.1″ W), and the other at Fazenda Santo Antonio (Saint Anthony Farm), city of Pires do Rio, state of Goiás (latitude 17° 12′ 38.8″ S, longitude 48° 18′ 24.9″ W). Cultivation was carried out in two Brazilian states with the aim of analysing the influence of two diverse geographical locations on the metabolic homeostasis of the species.

The 48 individuals of each group of matrices were cultivated from seedlings obtained by stem cuttings of two parent plants: PP-1 from the city of Ribeirão Preto, state of São Paulo; and PP-2 from the city of Pires do Rio, state of Goiás. PP-1 was the source of the seedlings used for the cultivation of the group of matrices for Ribeirão Preto while PP-2 was the seedling source for the group of matrices for Pires do Rio. A more detailed description of the cultivation of the groups of matrices of *T. diversifolia* is shown in the Supplementary Information (Method S1: Cultivation of *Tithonia diversifolia* by vegetative propagation).

The collections at both sites started in April 2012, three months after the *T. diversifolia* seedlings were transferred to the cultivation areas, with individuals in a young stage (with floral buds), and were consequently carried out monthly from May 2012 with individuals in an adult stage (bearing inflorescences), for a two-year period until March 2014. The collected material was separated according to plant parts (leaves, stems, roots, and inflorescences), packed in labelled paper bags, immediately frozen on dry ice (−78 °C) after collection, dried in freeze dryer and stored in a refrigerator at −20 °C until subjected to extraction, following an adapted standard operating procedure^19^.

Voucher specimens for each sampled population were deposited and identified at Herbarium SPF, Institute of Biosciences, University of São Paulo, under the responsibility of the curation of Dr. Renato de Mello Silva. Voucher numbers for each specimens were assigned as Sampaio #01 (samples from Pires do Rio-GO) and Sampaio #02 (samples from Ribeirão Preto-SP). All the samples and the voucher specimens were prepared according to the standard operating procedure for the access and shipment of component of genetic heritage, No. 010319/2013–1, as issued by the National Council for Scientific and Technological Development (*Conselho Nacional de Desenvolvimento Científico e Tecnológico –* CNPq), under authorization of Genetic Heritage Management Council (*Conselho de Gestão do Patrimônio Genético –* CGEN).

### Collection of soil samples and climate data

Commencing from April 2012, soil collection was performed every six months, following the standard procedure for soil analysis recommended by the Agronomic Institute of Campinas (*Instituto Agronômico de Campinas*, IAC). Soil samples were placed in labelled plastic bags (four replicates for each geographical location) and a 200 g aliquot was separated and sent for soil analysis of macro and micronutrients, including sulphur and aluminium composition^60^.

For all the plant collection areas covered in this study, climate data information between March 2012 and March 2014 was obtained from the available online climate database of the National Institute of Meteorology (*Instituto Nacional de Meteorologia –* INMET). Climate data included temperature (°C), humidity (%), solar radiation (kJ/m^2^) and rainfall (mm). For data analysis, monthly averages of temperature, humidity and radiation, and accumulated rainfall for each month were used.

### Preparation of crude extracts of *T. diversifolia*

To prepare the crude extracts used in this study, freeze-dried samples of different plant parts (leaves, stems, roots and inflorescences) were ground in a mill grinder (11 Basic IKA Model), and was sieved through a size 42 mesh (opening = 0.355 mm), packed in plastic microtubes and then stored at −20 °C.

From each collected sample, 100 mg aliquots were weighed into plastic microtubes (a total of 170 samples - 48 samples each of leaves, stems and roots, and 26 samples of inflorescences), 2 mL of 70% ethanol (v/v) were added and the extraction was performed in an ultrasonic bath for 20 min. After the extraction procedure, the material was centrifuged for 3 min at 13,000 rpm, the supernatant was filtered through a syringe filter (0.22 μm pore diameter), the filtrate was further cleaned-up by partitioning with *n*-hexane (95% HPLC grade) to remove excess of fats (fatty acids, waxes, etc.) and pigments, followed by centrifugation to separate the *n*-hexane partition which was then discarded. The fat-free plant extracts were dried in a centrifugal vacuum prior to metabolite variation analyses.

### Preparation of extracts for analysis by UHPLC-DAD-(ESI)-HRMS

For the preparation of the samples for UHPLC-DAD-(ESI)-HRMS analysis, the obtained extracts from the previous section were dissolved in water (Milli-Q)/acetonitrile (LC-MS grade) (7:3, v/v) at a concentration of 1 mg/mL then filtered through a syringe filter (0.22 μm). A solvent blank of water/acetonitrile (7:3), was also prepared in order to subtract any solvent interfering signals in the data processing step. A reference sample containing a mixture of extracts from various plant parts was used to align the chromatograms during data processing.

### Preparation of extracts for analysis by ^1^H NMR and J-resolved

A total of 170 *T. diversifolia* extracts was analysed by nuclear magnetic resonance spectroscopy (^1^H NMR and *J*-resolved) at the Strathclyde Institute of Pharmacy and Biomedical Sciences, Glasgow, UK. Access and sample shipment were in accordance with the Brazilian laws (Term of Authorization for Access and Shipment No. 010319/2013-1 issued by CNPq and approved by the letter COAPG No. 182/2014).

For NMR analysis, 5 mg of each of the sample were dissolved in 650 μL of methanol-d_4_/D_2_O (600:50) and transferred to 5 mm diameter 7″ NMR tubes.

### Analysis of the extracts by UHPLC-DAD-(ESI)-HRMS

The 170 extracts were analysed by UHPLC-DAD-(ESI)-HRMS Thermo Scientific^®^ Accela, equipped with Accela 1250 quaternary pumps, coupled to a Accela photodiode array detector and a Thermo Scientific^®^ Exactive Plus mass spectrometer with an Orbitrap^®^ analyzer.

Chromatographic analysis of the extracts was performed on an ACE^®^ analytical column (3.0 mm internal diameter × 150 mm length, 3 μm particle size) placed in an oven maintained at 35 °C using H_2_O with 0.1% formic acid as solvent A and acetonitrile with 0.1% formic acid as solvent B with the following gradient elution program; at 0 min, it started with 5% B which was increased to 20% B in 10 min , then to 25% B in 15 min to 45% B in 25 min, and to 100% B in 30 min, continued at 100% B to 38 min for washing and was equilibrated back to 5% B to 44 min at a solvent flow rate of 400 μL/min.

Electrospray ionization method was used for mass spectrometry under the following conditions: spray voltage (positive mode = 3.6 kV; negative mode = 3.2 kV); temperature of the capillary in positive mode at 300 °C and 320 °C in the negative mode. The analyzer used was an Orbitrap^®^.

### Analysis of the extracts by NMR

NMR was carried out for all the 170 *T. diversifolia* extracts on a 400 MHz Jeol-LA400 FT-NMR spectrometer system equipped with a 40TH5AT/FG probe (JEOL, Tokyo, Japan). The acquisition of the one-dimensional proton spectra (^1^H NMR) was performed by the pre-saturation pulse sequence using 16 scans per analysis. Subsequently, two-dimensional *J*-resolved NMR spectra were also acquired with 32 scans and 64 increments per scan. Data points were collected into a plot using spectral widths of 3.56 kHz for F2 (chemical shift axis) and 50 Hz for F1 (spin–spin coupling constant axis). The pre-saturation method was used to suppress the solvent signal during acquisition and the two-dimensional *J*-resolved spectra provided separation of overlapping chemical shift values (δ) against corresponding coupling constants (*J*) at different axes.

### Dereplication of the extracts by UHPLC-DAD-(ESI)-HRMS

The data obtained for the different *T. diversifolia* extracts, after analysis by UHPLC-DAD-(ESI)-HRMS, were used in the step of dereplication, based on the comparison of the values of accurate mass and the profiles of absorbance into UV/Vis for the peaks detected with in-house compound libraries, AsterDB Asteraceae Database, http://www.asterbiochem.org/asterdb) and TitDB (Tithonia Database), and the Dictionary of Natural Products (http://dnp.chemnetbase.com/).

### Data processing and multivariate analysis

The data obtained after analysis of the extracts by UHPLC-DAD-(ESI)-HRMS were pre-treated and pre-processed for multivariate analysis. MS data was acquired on switch mode. The obtained data were separated between positive and negative mode of ionization and converted to mzXML format with ProteoWizard 3.0.6002 package MSConvert software (Proteowizard Software Foundation). The sorted data was processed by MZmine 2.10 (MZmine 2 project) for peak detection, peak filtering, chromatogram construction, chromatogram deconvolution, isotopic peak grouping, chromatogram alignment, gap filling, and the search for adducts and peak identification using an in-house compounds database. The following MZmine parameters were used for the data processing: noise level at 10^6^; Lorentzian function for the peak shape; minimum peak height at 5 × 10^6^; and *m/z* tolerance at 0.002 *m/z* or 5.0 ppm.

The processed data were then exported as tables categorized according to peak areas, exact mass, and retention times for each sample extract, which were then subjected for multivariate statistical analysis.

The acquired 2D *J*-resolved spectrum were first tilted at 45° to remove the effect of constant couplings over the shaft with the chemical shift values then symmetrized. The spectra on the one-dimensional projection were extracted then processed following the same steps described for ^1^H spectra.

The data obtained by ^1^H NMR and *J*-resolved analyses were processed with MestReNova version 8 (Mestrelab Research S.L.^©^) prior to multivariate analysis. The ^1^H NMR spectra of respective extracts were stacked and processed in active spectrum mode. Pre-processing included baseline correction using the Whittaker-Smoother method, apodization was at a Gaussian function of 1 GB [Hz], normalization was on the highest signal equivalent to 100, smoothing was done with the Savitzky-Golay method, binning to the full spectrum used a value of 0.01 ppm for each bin, then subjected to a second normalization step to normalize the intensity of each bin.

The processed NMR data were exported as electronic tables containing the intensities of the signals for each chemical shift at 0.01 ppm intervals. Chemical shift values between 0.5 to 9.25 ppm and 0.5 to 8.9 ppm were used from the ^1^H and *J*-resolved spectral data, respectively. The solvent peaks for methanol-d_4_ and D_2_O were manually deleted in MS-Excel^®^.

The environmental data sets for the soil nutrients and climate from the two collection sites of *T. diversifolia* were processed according to the data type - data expressed as percentages (relative humidity and percentage of saturation for bases) and data not expressed as percentages (organic matter, levels of P, K, Ca, Mg, S, B, Cu, Fe, Mn and Zn, soil total acidity, cation exchange capacity and the sum of the bases). The environmental data expressed in percentages were transformed by the equation (1), while the other experimental data not expressed in percentages were calculated using the equation (2). For the soil pH, unprocessed data were used.


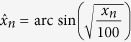


(1.) Transformation of variables by the arcsin method.


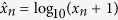


(2.) Logarithmic transformation for the variables.

The obtained processed and normalized spectral data from UHPLC-DAD-(ESI)-HRMS and ^1^H-NMR were combined using a data fusion method known as concatenation^39^. The data were divided into two blocks consisting of the LC-MS data block and ^1^H-NMR data block). Each block was scaled by the equation below (3). After the scaling procedure, both blocks were combined into one single data matrix which was subjected to multivariate analysis.


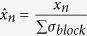


(3.) Equation of variable scaling for data fusion by concatenation method.

For the study of metabolite variation in *T. diversifolia*, after appropriate treatment of the separate and concatenated data obtained by UHPLC-DAD-(ESI)-HRMS and NMR against the climate and soil data, the results were analysed with CANOCO 4.5 (Biometris – Plant Research International^©^), OriginPro 9.6 (OriginLab Corporation^©^) and SIMCA-P (Umetrics^©^). The data were divided into two sets of variables: chemical (data obtained from the analysis of the extracts) and environmental variables (climate and soil data). These variables were used to perform multivariate analysis by unsupervised method through Principal Component Analysis (PCA) and Hierarchical Cluster Analysis (HCA) by the Ward’s method^61^ as well as a supervised method by Orthogonal Partial Least Squares Discriminant Analysis (OPLS-DA). These methods are ordering methods, with the aim of reducing the data set dimension to be able to explain the total variation of the system and clustering of the samples according to their chemical profile.

## Additional Information

**How to cite this article**: Sampaio, B. L. *et al*. Effect of the environment on the secondary metabolic profile of *Tithonia diversifolia*: a model for environmental metabolomics of plants. *Sci. Rep*. **6**, 29265; doi: 10.1038/srep29265 (2016).

## Supplementary Material

Supplementary Information
