# Supplementary material for: Effect of the environment on the secondary metabolic profile of Tithonia diversifolia: a model for environmental metabolomics of plants
Source: Sci Rep. 2016 Jul 7;6:29265. doi: 10.1038/srep29265 (PMC4935878; doi:10.1038/srep29265)
Supplement: Supplementary Information [file srep29265-s1.pdf]

Effect of the environment on the secondary metabolic profile of *Tithonia diversifolia*: a model for environmental metabolomics of plants.

Bruno Leite Sampaio[1,2+]; RuAngelie Edrada-Ebel[2*+]; Fernando Batista Da Costa[1+].

<u>**Supplementary Information**</u>

Method S1: Cultivation of *Tithonia diversifolia* by vegetative propagation

The two groups of matrices of *Tithonia diversifolia* used in this study were cultivated (*ex situ* condition) from seedlings obtained by vegetative propagation of cuttings from stems collected from two distinct parent plants (*in situ* condition) separated by a distance of approximately 500 km. Parent plant PP-1 was from the city of Ribeirão Preto, state of São Paulo while PP-2 was from the city of Pires do Rio, state of Goiás. Both counties are located within the Brazilian territory. The stem cuttings from PP-1 and PP2 were used to obtain the seedlings cultivated at Ribeirão Preto and Pires do Rio, respectively.

To obtain the seedlings of *T. diversifolia*, stem cuttings containing at least two axillary buds were transferred to plastic pots and partially covered with soil collected from the original cultivation sites (Garden of Medicinal Plants of the School of Pharmaceutical Sciences of Ribeirão Preto, University of São Paulo, Ribeirão Preto, and Fazenda Santo Antonio, county of Pires do Rio). Forty-eight pots of stem cuttings were prepared for each cultivation site. The method of vegetative propagation was chosen to obtain the seedlings of *T. diversifolia* due to the great capacity of this species to grow clonally[1], which ensures an intra-group genetic homogeneity to the specimens of both groups of matrices.

The pots containing the seedlings were kept outdoors, so that they could receive sunlight. Water was added once a day to ensure that the soil within the pots was always wet. The seedlings obtained from the stem cuttings were kept in pots for two months until complete rooting; and then were transferred to the soil of respective cultivation areas. Each

cultivation areas were divided into four quadrants, 12 seedlings of *T. diversifolia* were planted in each quadrant, totaling to 48 specimens per area.

The seedlings were transferred to the cultivation areas and were kept for three months under local environmental conditions to acclimatise. The first sample collection was done after the emergence of the floral buds (April 2012). However, inflorescence samples were obtained only from the second collection (May 2012), when they were full-grown.

Method S2: UHPLC-DAD-(ESI)-HRMS and NMR (J-resolved) data fusion by concatenation method

The data obtained by UHPLC-DAD-(ESI)-HRMS and *J*-resolved NMR were combined in one single data matrix using the concatenation method described by Forshed et al. (2007)[2] prior to multivariate statistical analysis.

The two sets of non-scaled data were divided in two groups according to the type of data (UHPLC-DAD-(ESI)-HRMS data – Block 1; and *J*-resolved NMR data – Block 2). Both data blocks were scaled by the formula described in Equation (1), such that x_n represents the chromatographic peak area value for UHPLC-DAD-(ESI)-HRMS data or signal intensity for *J*-resolved data for each observation of a given variable, and $\sum\sigma_{block}$ is the sum of all the standard deviations of each variable in each block. The scaled data ($\hat{x}_n$) were obtained by dividing the value of each peak area (Block 1) or signal intensity (Block 2) by the sum of standard deviations of the respective block.

$$\hat{x}_n = \frac{x_n}{\sum \sigma_{block}}$$

(1). Equation of variable scaling for data fusion by concatenation method.

After the scaling procedure, the data of the two blocks were arranged as a single data matrix (for each respective plant part), merging both blocks in the same data sheet on an MS Excel© software. The matrix obtained by the fusion of both type of data (LC-MS and NMR) was named as concatenated data matrix and it was used to perform multivariate analysis.

Table S1: Macro and micronutrients levels in soils of the cultivation sites in the states of Goiás (GO) and São Paulo (SP).

**Sampling**	**P***	**K****	**Ca****	**Mg****	**S***	**B***	**Cu***	**Fe***	**Mn***	**Zn***
GO-Apr/12	15,9	3,1	40,0	11,0	6,0	0,27	1,8	50,0	5,2	4,8
GO-Oct/12	12,0	2,9	32,0	11,0	6,0	0,24	1,3	23,0	4,9	2,2
GO-Apr/13	13,0	1,8	34,0	7,0	5,0	0,20	1,2	22,0	1,9	0,6
GO-Oct/13	24,0	3,2	27,0	9,0	5,0	0,22	2,0	38,0	5,7	2,3
SP-Apr/12	13,0	3,3	25,0	8,0	8,0	0,22	6,2	20,0	14,6	0,5
SP-Oct/12	12,0	2,8	27,0	9,0	7,0	0,24	4,1	10,0	10,7	0,4
SP-Apr/13	9,0	2,5	20,0	8,0	5,0	0,23	3,8	11,0	10,0	0,3
SP-Oct/13	7,0	2,6	22,0	7,0	6,0	0,20	5,2	14,0	13,6	0,6

*P, Fe, Mn, Cu, Zn, B e S expressed as mg/dm^3; **K, Ca, Mg expressed as mmolc/dm^3.

Table S2: Other indicators of the mineral composition of the soils of the cultivation sites in the states of Goiás (GO) and São Paulo (SP).

**Sampling**	**pH ($CaCl_2$)**	**OM***	**H+Al**	**CEC****	**BS*****
GO-Apr/12	5,8	29,0	19,0	73,0	74,0
GO-Oct/12	5,6	23,0	22,0	67,0	67,9
GO-Apr/13	6,0	22,0	20,0	63,0	68,1
GO-Oct/13	5,5	25,0	21,0	60,0	64,9
SP-Apr/12	5,2	30,0	31,0	67,0	54,0
SP-Oct/12	5,2	28,0	32,0	70,0	54,4
SP-Apr/13	5,1	27,0	34,0	65,0	47,5
SP-Oct/13	5,3	25,0	29,0	60,0	52,6

*OM expressed as g/dm^3; **CEC and H+Al expressed as mmolc/dm^3; BS expressed as percentage.
Legend: OM = Organic matter; H+Al = Value of potential acidity of soil; CEC = Cation exchange capacity; BS = Base saturation of soil.

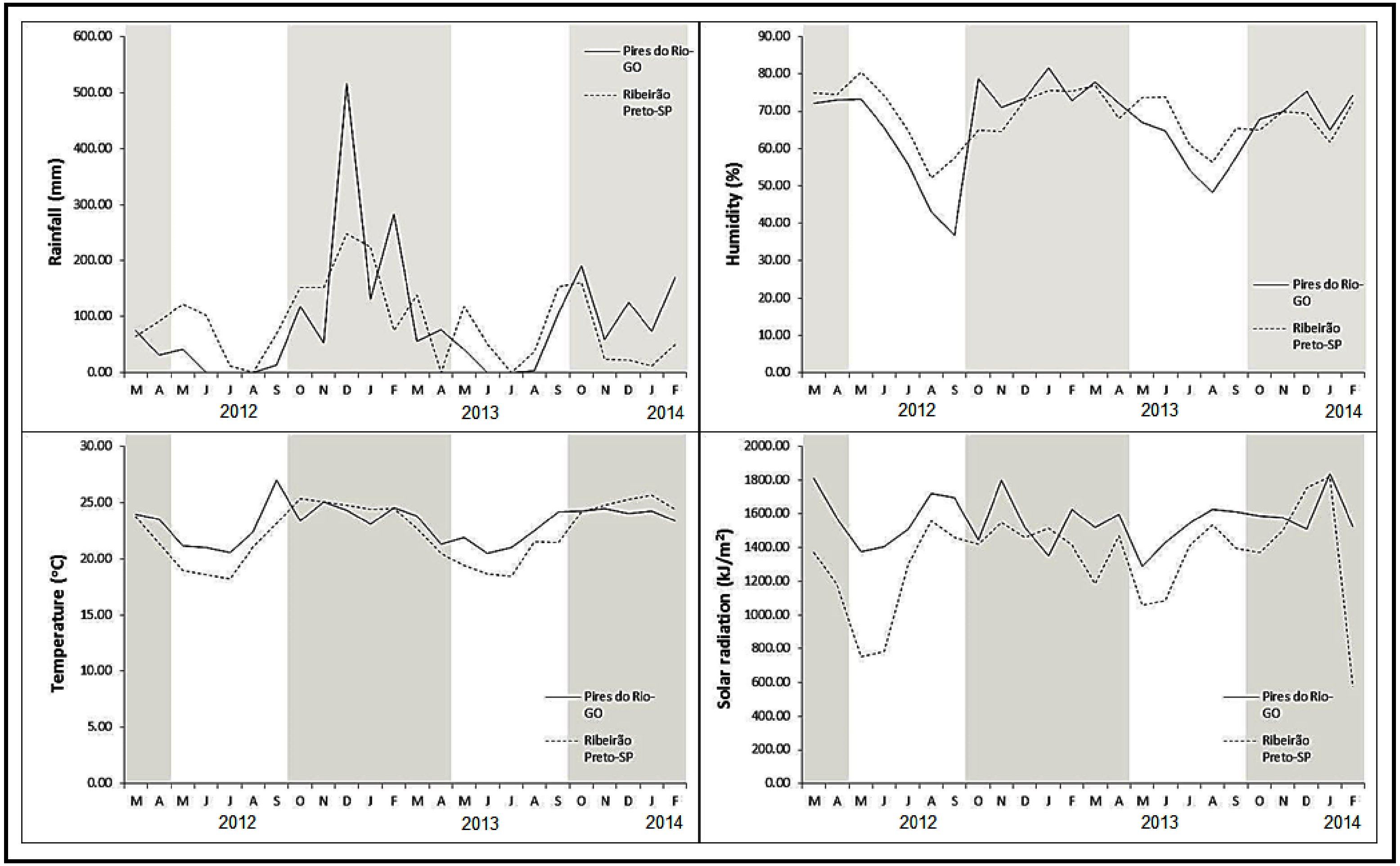

Figure S1: Climate data obtained from the both collection sites during the 24 months of study. Temperature, humidity and solar radiation were expressed as monthly average values and rainfall as accumulated rain per month. Continuous line = Pires do Rio-GO; Dashed line = Ribeirão Preto-SP. Gray areas in the graph indicate the rainy season and the white areas indicate the dry season.

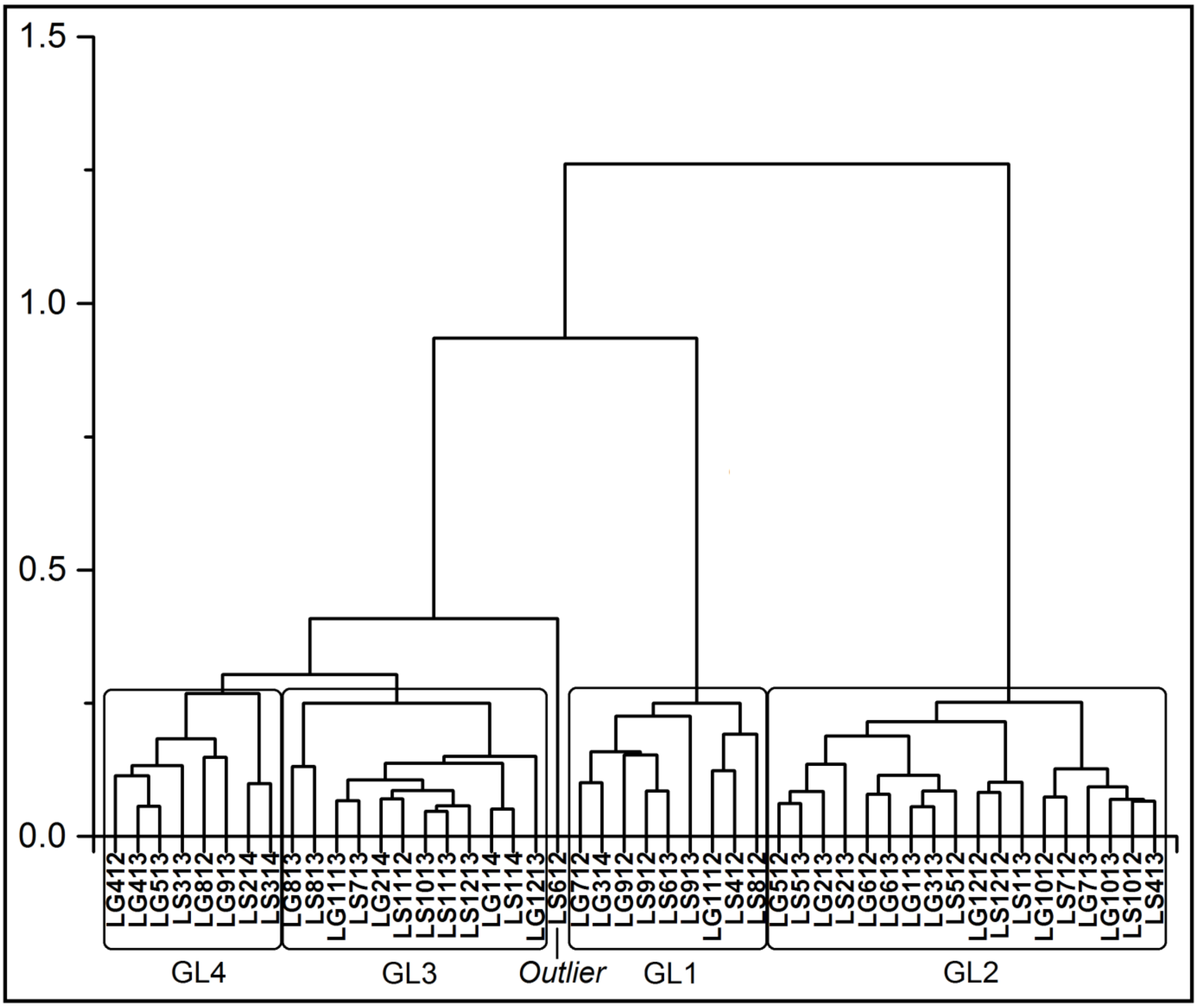

Figure S2: HCA dendrogram for concatenated data obtained from *T. diversifolia* leaf extracts. Box indicates grouping proposal. Legend: GL = Group of leaves extracts; LS = Leaves collected from São Paulo state; LG = Leaves collected from Goiás state; Number (eg. 112 for Jan 2012 or 1114 for Nov 2014)) represents the month (1 for January until 12 for December) and year (12 for 2012 until 14 for 2014).

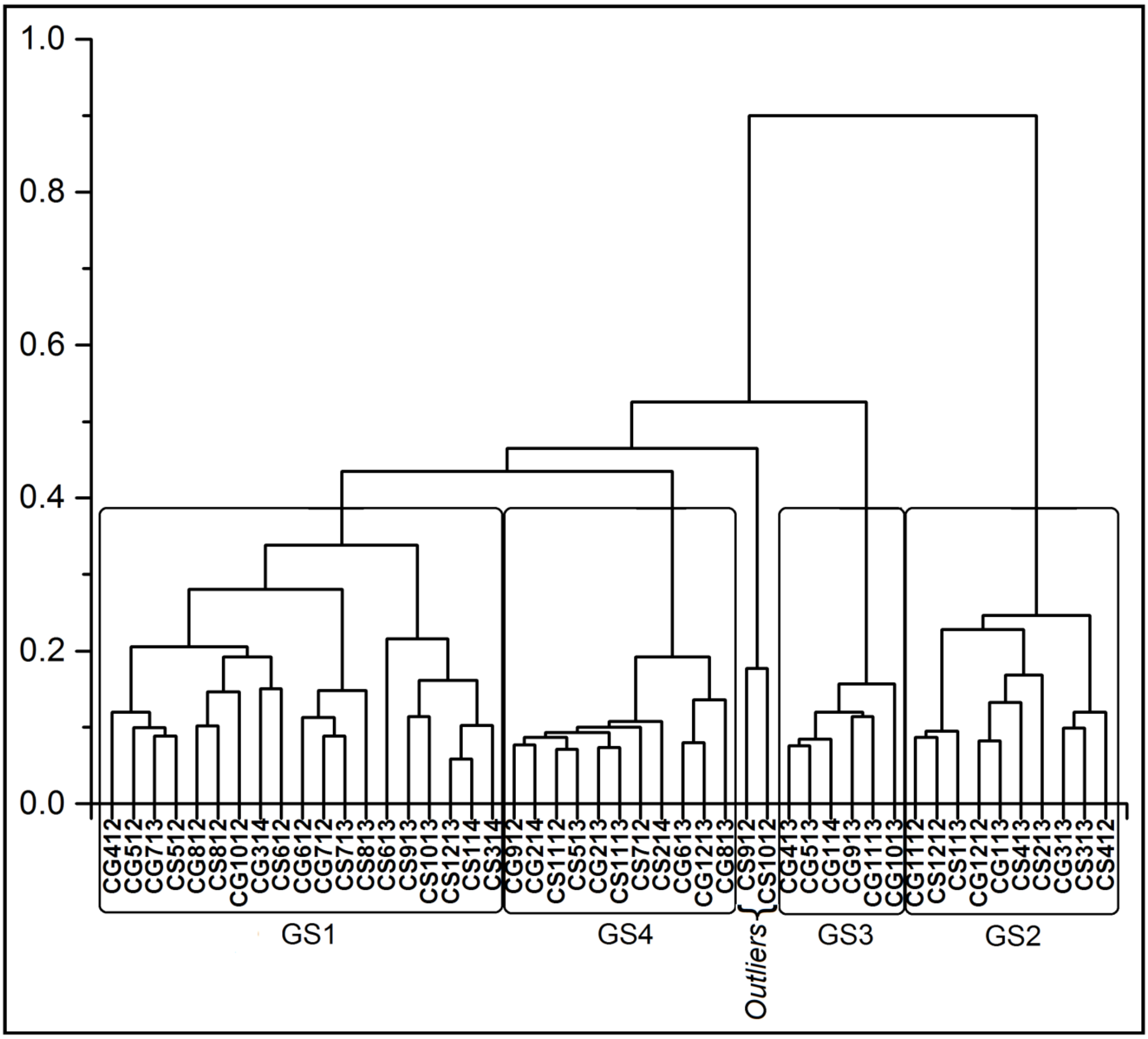

Figure S3: HCA dendrogram for the concatenated data obtained from *T. diversifolia* stem extracts. Box indicates grouping proposal. Legend: GS = Group of stems extracts; SS = Stems collected from São Paulo state; SG = Stems collected from Goiás state; Number (eg. 112 for Jan 2012 or 1114 for Nov 2014)) represents the month (1 for January until 12 for December) and year (12 for 2012 until 14 for 2014).

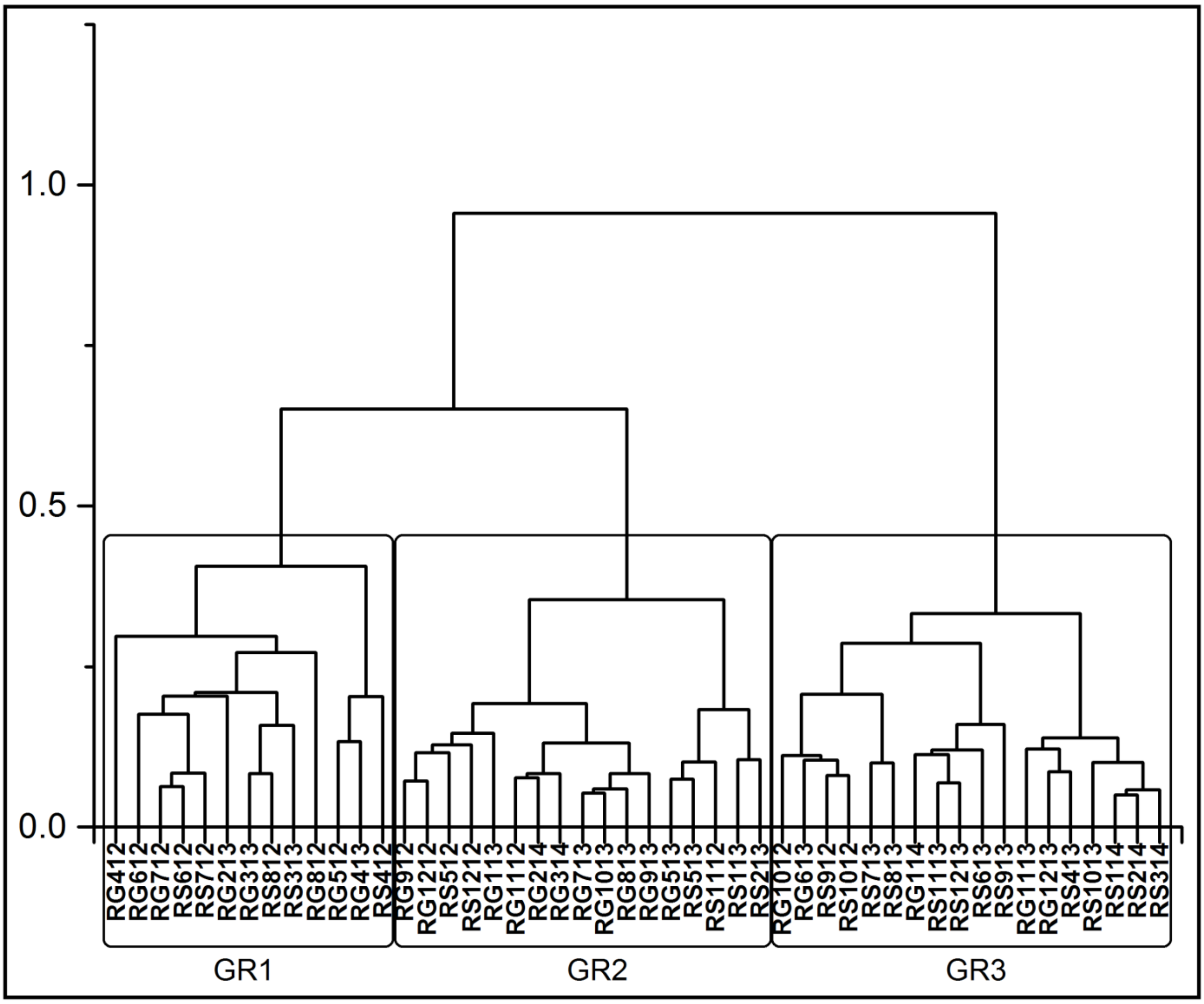

Figure S4: HCA dendrogram for the concatenated data obtained from *T. diversifolia* root extracts. Box indicates grouping proposal. Legend: GR = Group of roots extracts; RS = Roots collected from São Paulo state; RG = Roots collected from Goiás state; Number (eg. 112 for Jan 2012 or 1114 for Nov 2014)) represents the month (1 for January until 12 for December) and year (12 for 2012 until 14 for 2014).

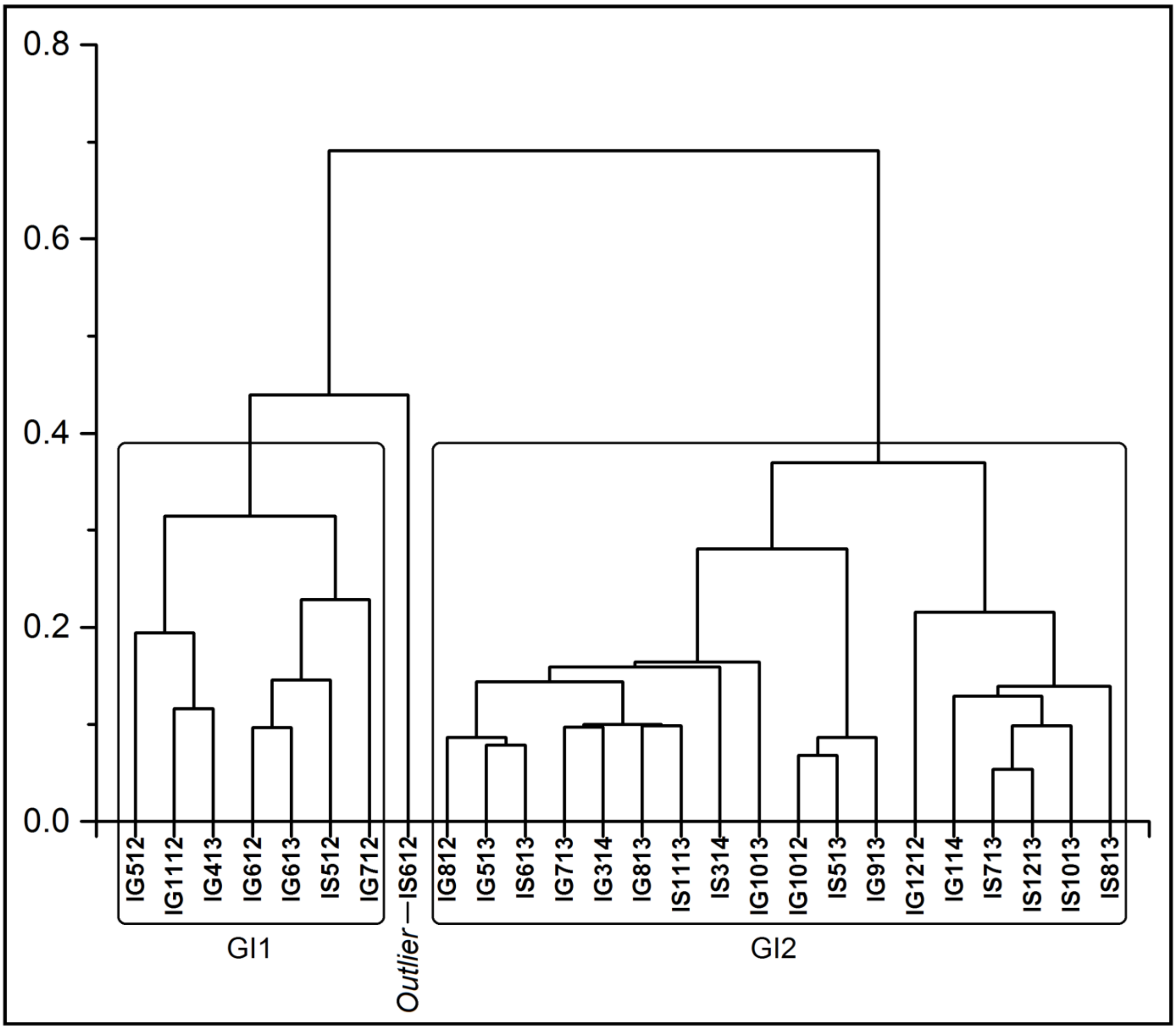

Figure S5: HCA dendrogram for the concatenated data obtained from *T. diversifolia* root extracts Box indicates grouping proposal. Legend: GI = Group of inflorescences extracts; IS = Inflorescences collected from São Paulo state; IG = Inflorescences collected from Goiás state; Number (eg. 112 for Jan 2012 or 1114 for Nov 2014)) represents the month (1 for January until 12 for December) and year (12 for 2012 until 14 for 2014).

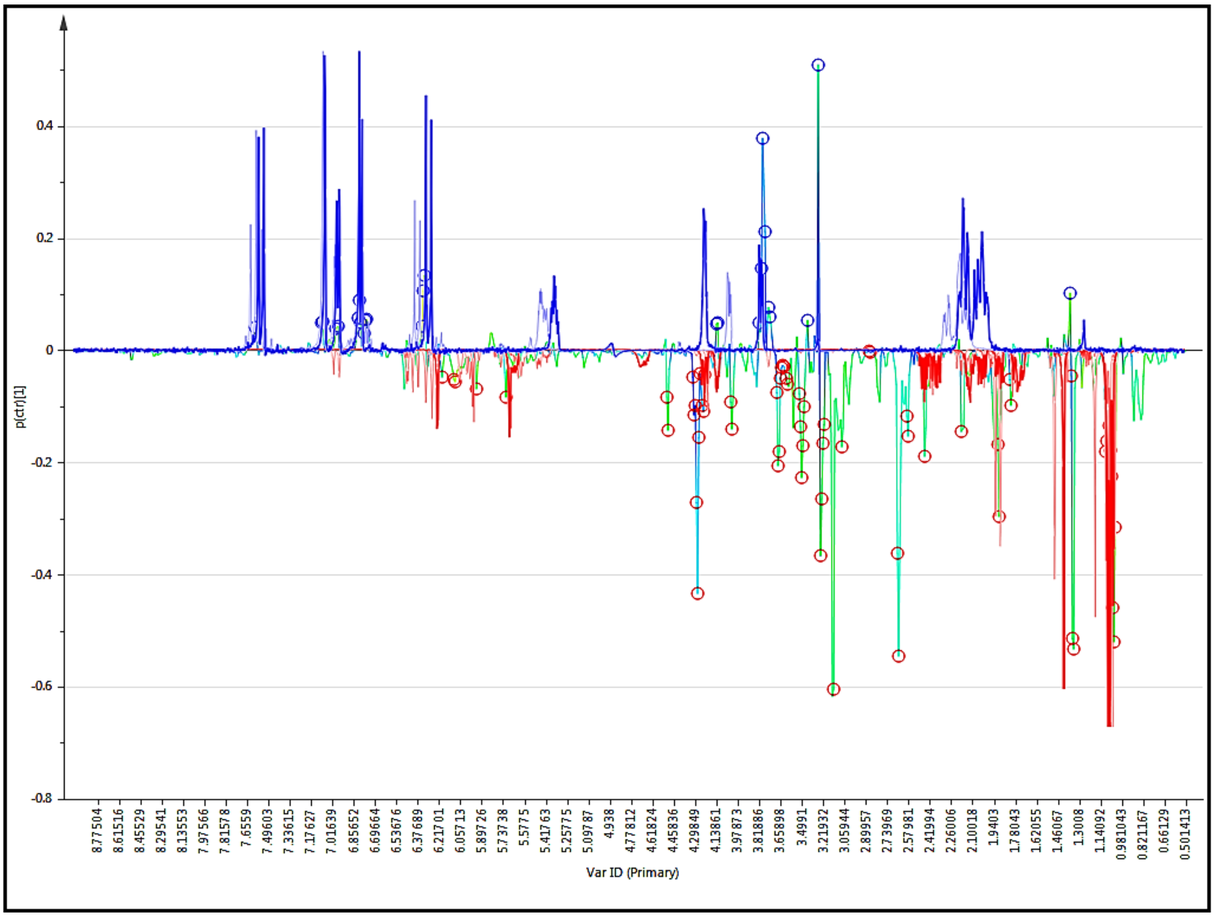

Figure S6: S-line plot for *J*-resolved data of leaf extracts overlaying the ^{1}H-NMR spectra of the major compounds for groups GL1 (red) and GL3 (blue). The circles indicate the discriminant chemical shifts for the two proposed groups. The spectra in red were obtained from sesquiterpene lactones tagitinin A, tagitinin C and tagitinin C epoxide, and the spectra in blue belong to caffeoylquinic derivatives chlorogenic acid and 3,5-O-dicaffeoylquinic acid.

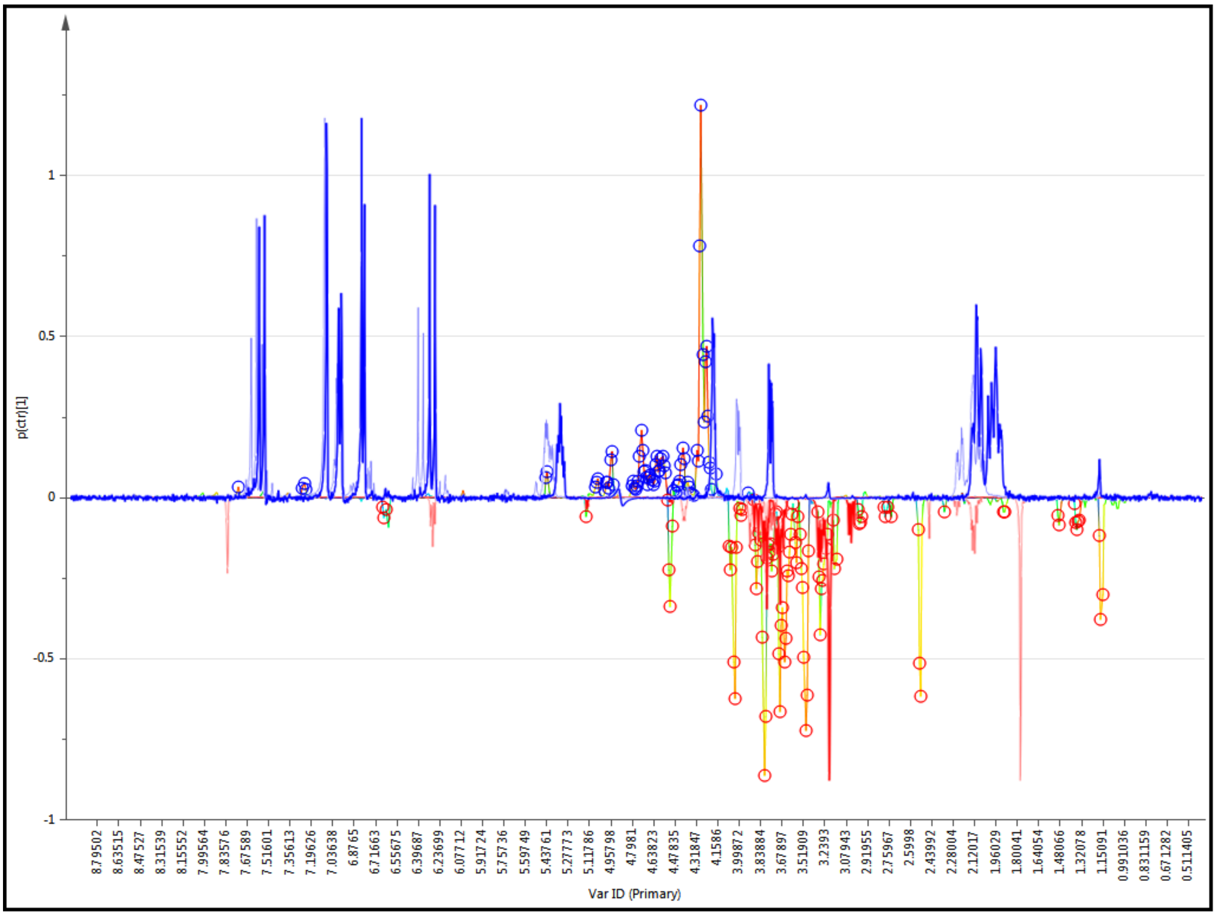

Figure S7: S-line plot for the *J*-resolved data of stem extracts overlaying the ^{1}H-NMR spectra of the major compounds for groups GS2 (red) and GS3 (blue). The circles indicate the discriminant chemical shifts for the two proposed groups. The spectrum in red represents the primary metabolite glucose and the spectra in blue belong to caffeoylquinic derivatives chlorogenic acid and 3,5-O-dicaffeoylquinic acid.

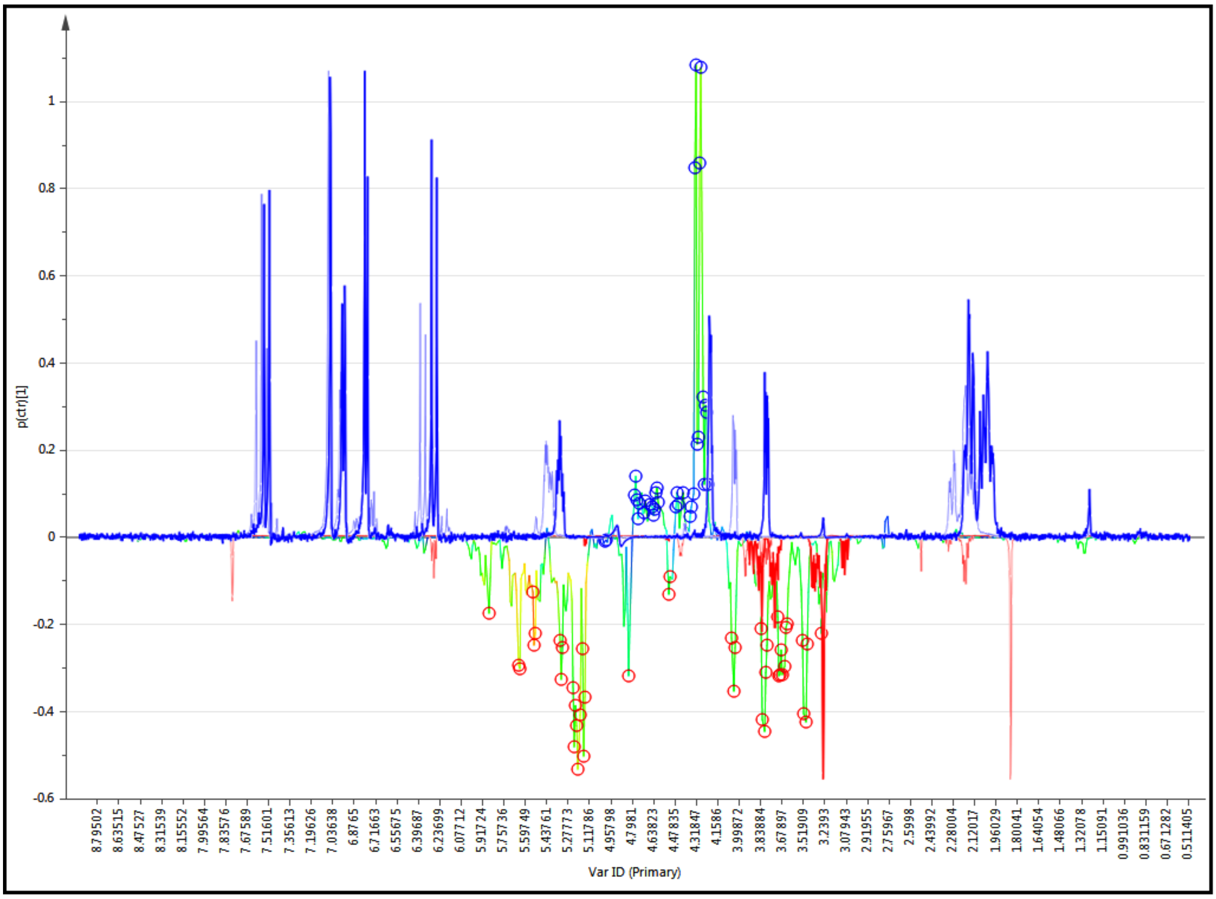

Figure S8: S-line plot for *J*-resolved data of root extracts overlaying the ^{1}H-NMR spectra of the major compounds for groups GR1 (red) and GR3 (blue). The circles indicate the discriminant chemical shifts for the two proposed groups. The spectra in red represent the primary metabolites glucose and thymidine, and the spectra in blue are caffeoylquinic derivatives, chlorogenic acid and 3,5-O-dicaffeoylquinic acid.

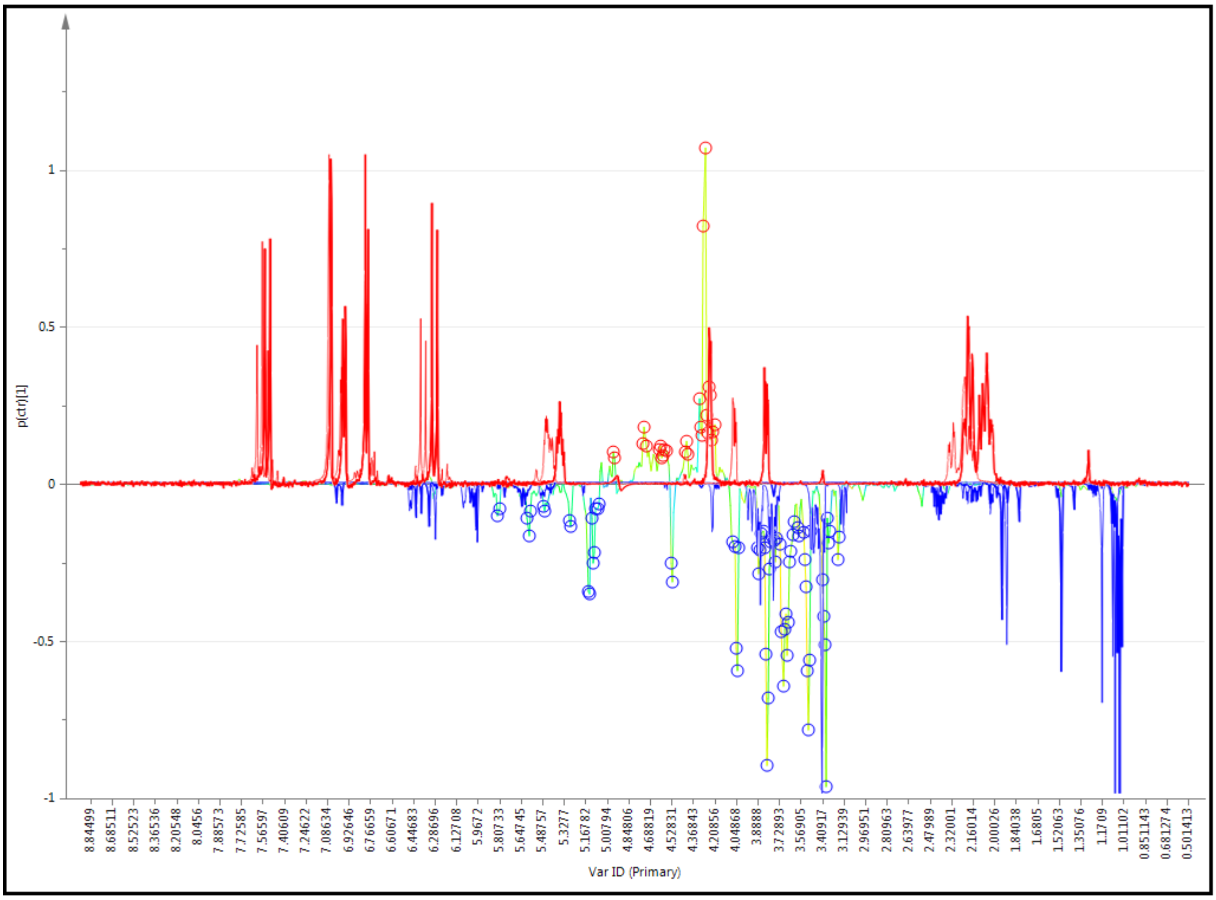

Figure S9: S-line plot for the J-resolved data of the extracts of inflorescences overlapped by the ^{1}H-NMR spectra of the main compounds for the groups GI1 (blue) and GI2 (red). The circles indicate the discriminant chemical shifts for the two proposed groups. The spectra in blue were obtained from the primary metabolite glucose and the sesquiterpene lactones tagitinin A, tagitinin C and tagitinin C epoxide, and the spectra in red from the caffeoylquinic derivatives chlorogenic acid and 3,5-O-dicaffeoylquinic acid.

Table S3: Result of the dereplication step performed with the extracts of *T. diversifolia* by UHPLC-DAD-(ESI)-HRMS.

ID	Molecular Formula	Monoisotopic Mass	Suggested metabolite class or compound	RT[1] (min)	Additional Information[2]	Plant Part
1	$C_6H_{12}O_7$	196.0575	Hamamelonic acid (*D*-form)	1.80	-	L; S; R; I
2	$C_5H_{13}O_7P$	216.0395	2-Methyl-1,2,3,4-butanetetrol; (2S,3R)-form, 4-Phosphate	1.80	Intermediate in the MEP pathway	L; S; R; I
3	$C_6H_{12}O_6$	180.0634	Sugar	1.84	-	L; S; R; I
4	$C_7H_{12}O_6$	192.0634	Quinic acid	1.84	-	L; S; R; I
5	$C_7H_{12}O_7$	208.0578	4-*O*-Methyl-*D*-glucuronic acid	1.99	-	L; S; R; I
6	$C_6H_8O_7$	192.0265	Citric acid	2.07	-	L; S; R; I
7	$C_7H_6O_4$	154.0266	Protocatechuic acid	6.05	Compared with a pure standard compound	L; S; I
8	$C_{16}H_{18}O_9$	354.0951	*Trans*-cinnamic acid derivative	6.30	UV = 324, 301sh, 247 nm	L; S; R; I
9	$C_7H_{12}O_6$	192.0367	Quinic acid	8.30	Resulting from the fragmentation of chlorogenic acid	L; S; R; I
10	$C_{16}H_{18}O_9$	354.0951	Chlorogenic acid (5-*O*-caffeoylquinic acid)	8.32	Compared with a pure standard compound	L; S; R; I
11	$C_{16}H_{18}O_9$	354.0951	*Trans*-cinnamic acid derivative	8.77	UV = 326, 300sh, 221 nm	L, S; R; I
12	$C_{24}H_{22}O_{14}$	534.1018	*Trans*-cinnamic acid derivative	9.77	UV = 323, 300sh, 248 nm	L, S; R; I
13	$C_9H_8O_4$	180.0415	Caffeic acid	9.80	Compared with a pure standard compound	L, S; R; I
14	$C_{14}H_{16}O_8$	312.0845	4-*O*-caffeoyl-2-*C*-methyl-*D*-threonic acid	11.90	Compared with a pure standard compound	L

15	$C_{27}H_{30}O_{16}$	610.1537	Rutin	12.90	Compared with a pure standard compound	I
16	$C_{21}H_{20}O_{12}$	464.0960	Isoquercitrin	13.75	UV = 364, 342sh, 260 nm	L; I
17	$C_{22}H_{22}O_{13}$	494.1060	Glycosylated flavonoid	14.00	UV = 354, 347, 293, 258 nm	L
18	$C_{25}H_{24}O_{12}$	516.1268	4,5-*O*-dicaffeoylquinic acid	14.80	Compared with a pure standard compound	L; S; R; I
19	$C_{25}H_{24}O_{12}$	516.1268	3,5-*O*-dicaffeoylquinic acid	15.40	Compared with a pure standard compound	L; S; R; I
20	$C_{33}H_{28}O_{17}$	696.1336	*Trans*-cinnamic acid derivative	15.50	UV = 328, 303sh, 247 nm	S; R
21	$C_{21}H_{20}O_{11}$	448.1010	Quercitrin	15.65	Compared with a pure standard compound	L; R; I
22	$C_{25}H_{24}O_{12}$	516.1273	Dicaffeoylquinic derivative	15.90	UV = 327, 300, 253 nm	L; S; R; I
23	$C_{24}H_{22}O_{15}$	550.0967	Glycosylated flavonoid	16.00	UV = 368, 348, 255 nm	L; I
24	$C_{25}H_{24}O_{12}$	516.1268	3,4-*O*-dicaffeoylquinic acid	16.60	Compared with a pure standard compound	L; S; R; I
25	$C_{33}H_{28}O_{17}$	696.6340	*Trans*-cinnamic acid derivative	16.90	UV = 328, 304sh, 245, 216	R
26	$C_{33}H_{28}O_{17}$	696.1323	*Trans*-cinnamic acid derivative	17.30	UV = 328, 303sh, 248	S; R
27	$C_{43}H_{38}O_{19}$	858.1656	*Trans*-cinnamic acid derivative	20.30	UV = 328, 304sh, 244	S; R
28	$C_{15}H_{10}O_{6}$	286.0480	Luteolin	20.90	UV = 346, 266, 254	L; S; I
29	$C_{15}H_{10}O_{7}$	302.0428	Quercetin	21.02	Compared with a pure standard compound	I
30	$C_{34}H_{37}N_{3}O_{7}$	599.2637	Spermidine; *N′*-(3,4-Dihydroxycinnamoyl), *N,N′′*-	21.10	UV = 306, 294	I

			bis(4-hydroxycinnamoyl)			
31	$C_{16}H_{12}O_7$	316.0583	Nepetin	21.20	UV = 346, 271, 255	L; S; I
32	$C_{16}H_{12}O_7$	316.0583	Flavonoid	21.30	UV = 346, 269, 256 nm	L; S; I
33	$C_{19}H_{26}O_7$	366.1679	Tagitinin B or 4,8,10-Trihydroxy-3-oxo-11(13)-guaien-12,6-olide; (1α,4β,5α,6α,8β,10β)-form, 8-O-(2-Methylpropanoyl)	21.30	-	L; S; I
34	$C_{19}H_{28}O_7$	368.1835	Tagitinin A ou 2α-hydroxytirotundin	21.80	-	L; S; I
35	$C_{34}H_{37}N_3O_6$	583.2682	Spermidine; *N,N′,N″*-Tris(4-hydroxy-*E*-cinnamoyl)	22.40	UV = 306, 294	I
36	$C_{19}H_{28}O_7$	368.1835	Tagitinin A or 2α-hydroxytirotundin	22.70	-	L; S; I
37	$C_{19}H_{26}O_7$	366.1679	Tagitinina B or4α,10α-dihydroxy-8β-isobutyroyloxy-3-oxoguai-11(13)-en-6α,12-olide	22.70	-	L; S; I
38	$C_{46}H_{50}N_4O_9$	802.3588	Monocaffeoyl-tri-p-coumaroyl spermine	23.05	UV = 305, 297	I
39	$C_{15}H_{10}O_5$	270.0525	Apigenin	23.45	UV = 338, 266 nm	L
40	$C_{16}H_{12}O_6$	300.0634	Hispidulin	23.80	UV = 335, 273 nm	L; S; R
41	$C_{46}H_{50}N_4O_8$	786.3629	Spermine; *N1,N5,N10,N14*-Tetrakis(4-hydroxy-E-cinnamoyl)	24.00	UV = 305, 297	I
42	$C_{18}H_{32}O_5$	328.2252	Phytoprostane *F1* type I	24.13	UV = 203 nm	L; S; I
43	$C_{19}H_{26}O_7$	366.1679	Tagitinin B or 4α,10α-dihydroxy-8β-isobutyroyloxy-3-oxoguai-11(13)-en-6α,12-olide	24.77	-	L; S; I

44	$C_{19}H_{24}O_6$	348.1573	Tagitinin C	24.90	Compared with a pure standard compound	L; S; I
45	$C_{19}H_{26}O_6$	350.1729	Tagitinin E ou diversifolin	24.90	-	L; S; I
46	$C_{20}H_{28}O_7$	380.1835	2-*O*-methyltagitinin B ou 3,10-Epoxy-1,3,8-trihydroxy-4,11(13)-germacradien-12,6-olide; (1β,3α,4Z,6α,8β)-form, 3-Me ether, 8-O-(2-methylpropanoyl)	25.30	-	L; S; I
47	$C_{12}H_{20}O_4$	228.1359	Hydroxylated fatty acid	25.30	-	L
48	$C_{18}H_{34}O_5$	330.2409	Hydroxylated fatty acid	25.50	-	L; S; R; I
49	$C_{19}H_{26}O_7$	366.1680	STL	26.50	-	L; I
50	$C_{20}H_{26}O_6$	362.1729	STL	27.30	-	L; S; I
51	$C_{20}H_{26}O_6$	362.1731	STL	27.60	UV = 223 nm	L; I
52	$C_{18}H_{32}O_4$	312.2303	Hydroxylated fatty acid	30.40	UV = 224 nm	S; R; I
53	$C_{18}H_{30}O_3$	294.2198	Fatty acid	30.80	-	L; R; I
54	$C_{27}H_{46}O_9$	514.3142	Triterpene or fatty acid	31.30	UV = 227 nm	L; R
55	$C_{20}H_{40}O_4$	344.2926	Fatty acid	34.60	-	L; R. I

[1]RT: Retention time of the metabolites in minutes. [2]Additional Information: Additional data about the metabolites useful for the dereplication step, such as the maximum absorbance in the UV, metabolites identified by comparison with a pure standard compound and other information.

References

1. Sun, W., Chen, G. & Wang, S. Characteristics of *Tithonia diversifolia*: an alien invasive plant in Yunnan, south-west China. in *3rd Glob. Bot. Gard. Congr.* **2,** 1–7 (Botanic Gardens Conservation International, 2007).
2. Forshed, J., Idborg, H. & Jacobsson, S. P. Evaluation of different techniques for data fusion of LC/MS and 1H-NMR. *Chemom. Intell. Lab. Syst.* **85,** 102–109 (2007).
